# Intermittent parathyroid hormone treatment affects the bone structural parameters and mechanical strength of the femoral neck after ovariectomy-induced osteoporosis in rats

**DOI:** 10.1186/s12938-022-00978-9

**Published:** 2022-01-29

**Authors:** Shun-Ping Wang, Ying-Ju Chen, Cheng-En Hsu, Yung-Cheng Chiu, Ming-Tzu Tsai, Jui-Ting Hsu

**Affiliations:** 1 Department of Orthopaedics, Taichung Veterans General Hospital, Taichung, 407 Taiwan; 2 Sports Recreation and Health Management Continuing Studies-Bachelor’s Degree Completion Program, Tunghai University, Taichung, 407 Taiwan; 3 Department of Post‐Baccalaureate Medicine, College of Medicine, National Chung Hsing University, Taichung, 402 Taiwan; 4 Bachelor Program in Health Care and Social Work for Indigenous Students, College of Humanities and Social Sciences, Providence University, Taichung, 433 Taiwan; 5 School of Medicine, China Medical University, Taichung, 404 Taiwan; 6 Department of Orthopedic Surgery, China Medical University Hospital, Taichung, 404 Taiwan; 7 Department of Biomedical Engineering, Hungkuang University, Taichung, 433 Taiwan; 8 School of Dentistry, College of Dentistry, China Medical University, 91 Hsueh-Shih Road, Taichung, 404 Taiwan; 9 Department of Bioinformatics and Medical Engineering, Asia University, Taichung, 413 Taiwan

**Keywords:** Femoral neck, Parathyroid hormone, Microcomputed tomography, Cortical bone, Cancellous bone

## Abstract

**Background:**

Menopause-induced decline in estrogen levels in women is a main factor leading to osteoporosis. The objective of this study was to investigate the effect of intermittent parathyroid hormone (PTH) on bone structural parameters of the femoral neck in ovariectomized rats, in addition to correlations of maximum fracture force.

**Methods:**

Fifteen female Wister rats were divided into three groups: (1) control group; (2) ovariectomized (OVX) group; and (3) OVX  +  PTH group. All rats were then killed and the femurs extracted for microcomputed tomography scanning to measure volumetric bone mineral density (vBMD) and bone structural parameters of the femoral neck. Furthermore, the fracture forces of femoral neck were measured using a material testing system.

**Results:**

Compared with the control and OVX  +  PTH groups, the OVX group had significantly lower aBMD, bone parameter, and mechanical strength values. A comparison between OVX and OVX  +  PTH groups indicated that PTH treatment increased several bone parameters. However, the OVX  +  PTH groups did not significantly differ with the control group with respect to the bone structural parameters, except for trabecular bone thickness of cancellous bone, which was greater. In addition, among the bone structural parameters, the CSA and BSI of cortical bone were significantly correlated with the maximum fracture force of the femoral neck, with correlations of, respectively, 0.682 (*p*  = 0.005) and 0.700 (*p*  = 0.004).

**Conclusion:**

Intermittent PTH helped treat ovariectomy-induced osteoporosis of cancellous bone and cortical bone in the femoral necks of rats. The ability of the femoral neck to resist fracture was highly correlated with the two parameters, namely cross-sectional area (CSA) and bone strength index (=  vBMD  ×  CSA), of cortical bone in the femoral neck and was less correlated with aBMD or other bone structural parameters.

## Background

Osteoporosis is a common and serious health issue globally [1–4]. Osteoporosis is serious because continual bone loss results in bone fragility, which, in turn, leads to osteoporotic fracture and subsequent severe pain and difficulty in movement. This decreases the patient’s quality of life. Operation may even be required in some cases, which carries inherent risks of surgery, the discomfort of anesthesia, greater medical expenditure, and a greater burden on the public health-care system [5]. Postmenopausal osteoporosis is common among postmenopausal women; this is because of decreased estrogen levels from decreased ovary function, which results in considerable bone loss, subsequently decreased bone mineral density, and subsequent fragile fractures [6]. According to the literature, approximately 30% of postmenopausal women are affected by osteoporosis, and approximately 40% of this 30% will have experienced one or more fragility fractures in their remaining lifetime [7–9].

Specifically, hip fractures are a devastating type of osteoporotic fracture, with approximately 1.6 million patients worldwide every year. The morbidity risk of hip fracture has increased with the rapid aging of the world population. Hip fracture leads to disability, and it has a mortality that is three to four times higher than that in the general population [10]. Furthermore, the mortality of hip fracture 1 year after surgery ranges from 16.6 to 30% [11–13]. Half of those patients who survive from a hip fracture lose their functional independence, and a third of them eventually become totally dependent [14], which greatly increases the burden on society [15]. Therefore, osteoporosis-related hip fractures, which are characterized by poor functional outcomes and a high mortality rate, should be prevented through interventions such as exercise and medication.

At present, osteoporosis can be treated by two types of commonly available drugs. The first is antiresorptive drugs, which are first-line drugs whose main function is to suppress osteoclasts. Among antiresorptive drugs, bisphosphonate drugs are the most common. Bisphosphonate drugs can significantly improve bone mineral density in the hips and vertebra body, which, in turn, decreases the probability of fracture [16]. However, the long-term use of bisphosphonate drugs overly suppresses osteoclast function and may be complicated with atypical femoral fractures, osteonecrosis of the jaw (ONJ), gastrointestinal intolerance, acute phase reaction (an influenza-like syndrome) as well as atrial fibrillation [17, 18]. The second type is anabolic drugs, among which parathyroid hormone medications are a specific type. Teriparatide [PTH (1–34), Forteo; Eli Lilly] is a segment of recombinant human PTH peptide. Intermittent teriparatide can effectively treat osteoporosis by (1) increasing the number of osteoblasts, stimulating their activity, and decreasing their apoptosis [19] and (2) increasing bone mass in the lumbar vertebra and long bones, increasing trabecular bone thickness, and bone mineral density in the hips or spine [20].

Bone remodeling, regulated by systemic continuous or intermittent PTH administration, influences the bone strength via altering bone quality including bone turnover, microarchitecture and morphology in addition to bone mineral density (BMD) [21–23]. Although many previous studies have elucidated that the provision of intermittent PTH contributes greatly to treating osteoporosis, these animal studies, have focused on changes of BMD or trabecular structures in vertebra body, tibia, or distal femoral metaphysis [24–26]; the effects of this anabolic agent on femoral neck were still not very clarified. Furthermore, the hormone-related bone remodeling has site-specific changes that are varied in different skeletal areas [27, 28]. The specific features make it difficult to assume the anabolic effects on other skeletal sites, either trabecular or cortical bone, to femoral neck. Therefore, the proximal femur of the OVX rat might be more clinically relevant to human hip fracture than other skeletal sites (i.e., proximal tibia or distal femur) for preclinical testing of therapeutic agents on OVX-related estrogen-deficiency bone loss. There is still a paucity of evidences on the preventive effects of intermittent PTH used in “early” menopausal stage for the prevention of bone deteriorations, in terms of bone density, microstructural changes and bone strength of femoral neck.

Thus, this study examined how intermittent PTH treatment affects cortical and cancellous bone structural parameters of the femoral neck in ovariectomized rats and how those parameters correlate with mechanical strength. To do so, a material testing system was used to measure the maximum fracture force of the femoral neck and to explore the correlation between such mechanical strength and the bone structural parameters assessed with DXA and micro-CT.

## Results

### Effect on mechanical strength and bone structure parameters by OVX and intermittent parathyroid hormone treatment

**Fig. 1:**
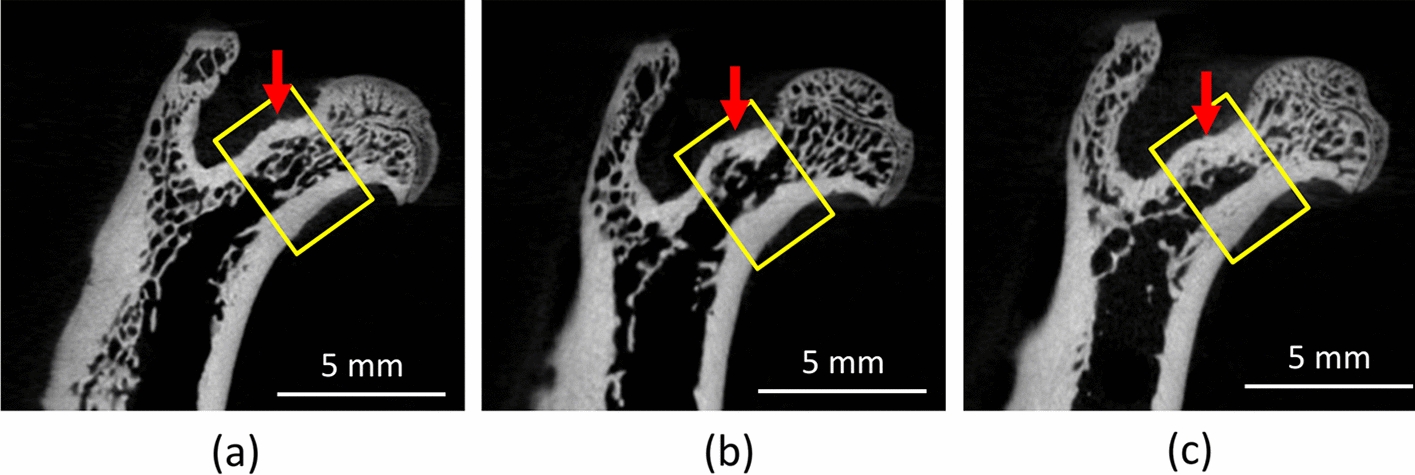
Coronal**-**sectional micro-CT images for the **a** control, **b** OVX, and **c** OVX  +  PTH groups. The images were obtained from one representative sample, and the yellow rectangle depicts the region of interest (ROI) for the measurement of the femoral neck. Thinning of cortical bone (red arrow) after OVX and regaining of thickness in the PTH treatment group

Table 1 lists the data on the aBMD, bone parameters of the femoral necks and mechanical strength of the three groups of rats. Compared with the control and OVX  +  PTH groups, the OVX group had significantly less mechanical strength of the femoral neck. Although the median of mechanical strength for the control group was 10.3% greater than that for the OVX  +  PTH group, this difference was not significant. In addition, compared with the control and OVX  +  PTH groups, the OVX group had a significantly less aBMD of the femoral neck. In essence, the femoral neck of the OVX group was the most cancellous, as revealed in 2D cross-sectional images and particularly for the cortical bone tissue, whereas the difference between the control and OVX  +  PTH groups was small (Fig. 1).

**Table 1:** Maximum fracture force, bone mineral density, and cortical and cancellous bone structure parameters of the femoral neck for the three groups

	Parameters (unit)	Value	Control	OVX	OVX + PTH	*p*^†^
Density	aBMD (g/cm^2^)	Median^d^	146.0^a^	139.0^b^	147.0^a^	0.306
		IQR	16.5	12.0	15.0	
		Mean	144.2	137.4	143.8	
		SD	8.5	5.6	7.5	
Cancellous bone	BV/TV (%)	Median^d^	56.957^ab^	49.977^a^	62.111^b^	0.065
		IQR	10.843	21.926	12.542	
		Mean	55.408	46.699	62.699	
		SD	5.356	11.525	6.196	
	TbTh (mm)	Median^d^	0.136^a^	0.128^a^	0.155^b^	0.008
		IQR	0.017	0.030	0.008	
		Mean	0.136	0.127	0.155	
		SD	0.009	0.015	0.004	
	TbSp (mm)	Median^d^	0.162^a^	0.170^a^	0.135^a^	0.164
		IQR	0.022	0.088	0.042	
		Mean	0.160	0.172	0.135	
		SD	0.011	0.040	0.020	
	TbN (1/mm)	Median^d^	4.140^a^	3.587^a^	4.076^a^	0.357
		IQR	0.335	1.278	0.679	
		Mean	4.062	3.617	4.031	
		SD	0.156	0.638	0.341	
Cortical bone	CtTh (mm)	Median^d^	0.451^a^	0.513^a^	0.520^a^	0.326
		IQR	0.140	0.055	0.050	
		Mean	0.477	0.514	0.513	
		SD	0.075	0.029	0.028	
	vBMD (g/cm^3^)	Median^d^	1.076^a^	1.060^a^	1.071^a^	0.402
		IQR	0.065	0.020	0.030	
		Mean	1.058	1.055	1.068	
		SD	0.037	0.009	0.015	
	CSA (mm^2^)	Median^d^	3.016^ab^	2.618^a^	2.852^b^	0.018
		IQR	1.076	0.127	0.943	
		Mean	3.265	2.618	3.173	
		SD	0.617	0.067	0.559	
	BSI	Median^d^	3.246^a^	2.763^b^	3.084^a^	0.013
		IQR	0.997	0.173	0.956	
		Mean	3.433	2.762	3.388	
		SD	0.524	0.089	0.593	
Mechanical strength	Maximum fracture force (*N*)	Median^d^	114.1^a^	80.6^b^	102.3^a^	0.046
		IQR	30.2	21.1	24.2	
		Mean	108.5	84.3	104.2	
		SD	15.2	12.8	12.3	

*aBMD* areal bone mineral density; *BV/TV *bone volume/total volume; *TbTh* trabecular bone thickness; *TbSp *trabecular bone separation; *TbN *trabecular bone number; *CtTh *cortical bone thickness; *vBMD *volumetric bone mineral density; *CSA *cross-sectional area; *BSI *bone strength index (vBMD  ×  CSA); *IQR *interquartile range; *OVX *ovariectomy; *PTH *parathyroid hormone

^d^Mann–Whitney exact tests were conducted for post hoc pairwise comparisons; medians in the same row with the same letter (a, b, or c) do not significantly differ

^†^Kruskal–Wallis test

The following are the results for the four cancellous bone parameters. BV/TV did not significantly differ between the control and OVX groups and between the control and OVX  +  PTH groups; however, BV/TV was significantly greater in the OVX  +  PTH group than in the OVX group. TbSp and TbN did not significantly differ between the three groups. TbTh was greater in the OVX  +  PTH group than in the control and OVX groups.

The following are the results for the four cortical bone parameters. CtTh and vBMD did not significantly differ between the three groups. CSA did not significantly differ between the control and OVX groups or between the control and OVX  +  PTH groups; however, CSA was significantly greater in the OVX  +  PTH group than in the OVX group. BSI did not significantly differ between the control and OVX  +  PTH groups; BSI in both these groups was greater than that in the OVX group.

### Correlations of aBMD and cortical and bone parameters with maximum fracture force of the femoral neck

According to Table 2, the aBMD and trabecular bone structural parameters were not significantly correlated with the maximum fracture force of the femoral neck (*p*  > 0.05). Moreover, among the cortical bone parameters, CtTh and vBMD were not correlated with the maximum fracture force of the femoral neck (*p*  > 0.05). However, both CSA (0.682, *p*  = 0.005) and BSI (0.700, *p*  = 0.004) were highly correlated with the maximum fracture force of the femoral neck. In addition, no significant correlation was found between the aBMD and vBMD (*r*  = − 0.213, *p*  = 0.445).

**Table 2:** Correlations of aBMD and bone structural parameters with maximum fracture force of femoral neck in terms of Spearman’s correlation coefficient (*r*) and probability (*p*)

Parameter (unit)	Spearman’s correlation of the parameters with maximum fracture force of femoral neck		Note
	*r*	*p*	
Bone mineral density			
aBMD	0.469	0.078	NC
Cancellous bone			
BV/TV (%)	0.425	0.114	NC
TbTh (mm)	0.257	0.355	NC
TbSp (mm)	− 0.132	0.638	NC
TbN (mm^−1^)	0.504	0.056	NC
Cortical bone			
CtTh (mm)	− 0.486	0.066	NC
vBMD (g/cm^3^)	0.027	0.924	NC
CSA (mm^2^)	0.682	0.005	High correlation
BSI	0.700	0.004	High correlation

*BV/TV* bone volume/total volume; *TbTh* trabecular bone thickness; *TbSp* trabecular bone separation; *TbN* trabecular bone number; *CtTh* cortical bone thickness; *vBMD* volumetric bone mineral density; *CSA* cross-sectional area; *BSI* bone strength index (vBMD  ×  CSA); *NC* no correlation

## Discussion

Osteoporosis has become more prevalent as societies have aged. Patients with osteoporosis face a higher risk of fractures and a decreased quality of life. Studies have suggested that in addition to antiresorptive drugs with bisphosphonates, anabolic drugs, such as those in intermittent PTH treatment, effectively treat osteoporosis. However, studies have mostly investigated the vertebra body or long bone [29–32]. Findings on how intermittent PTH treatment improves (1) the femoral neck bone structure or (2) the resistance of the femoral neck against fractures remain inadequate. This study used micro-CT to explore ovariectomy-induced osteoporosis in rats. Specifically, this study focused on how intermittent PTH treatment changed the cortical bone and cancellous bone structures of the femoral neck and how cortical bone and cancellous bone parameters were correlated with the ability of the femoral neck to resist fracture. The results indicated that intermittent PTH contributed to treating ovariectomized-induced osteoporosis in the cancellous bone and in the cortical bone of the femoral neck of rats. Femoral neck fracture strength was highly correlated with two parameters of the femoral neck (CSA and BSI) and no correlated with other BMD or bone structural parameters.

Previous studies on osteoporosis have mostly used rats or rabbits for animal experiments [33, 34], and the use of female rats has been particularly common in studies on ovariectomy to simulate menopause-induced osteoporosis in women [35]. A study suggested that rats reach sexual maturity at approximately 6 weeks old [33]. Thus, this study adopted female rats, which were bilaterally ovariectomized at the age of 8 weeks. Teriparatide, hPTH (1–34), is an effective anabolic agent for treating osteoporosis and daily use for 18 months has been recommended to treat osteoporosis in menopausal women. However, the optimal duration of its therapeutic effect on the bones of the ovariectomized rats has no consensus. The period of the intermittent PTH was varied from 6 to 16 weeks to evaluate the effects on bones in the previous studies [36–40]. The 12 weeks of teriparatide use for evaluating the bone changes in the OVX animal model was accepted and commonly used [36, 37, 40]. In this study, we adopted 12-week teriparatide injection to prevent the bone loss from the ongoing estrogen deficiency-related bone deteriorations after OVX. The rats of the treated group were treated with intermittent PTH for 12 weeks, and the corresponding changes in bone mineral density in the femoral neck, in bone structural parameters, and in fracture strength were measured.

This study first adopted DXA, the most commonly used method in clinical practice, to measure bone mineral density. However, DXA can only measure aBMD in two dimensions, whereas bone structure is in three dimensions; two dimensional aBMD measurements cannot completely represent bone strength. This study also used micro-CT to measure the cortical- and cancellous-bone structural parameters of the femoral neck. In general, micro-CT is the gold standard for estimating bone morphology and the microstructure. Micro-CT can be used to measure many parameters of the trabecular bone microarchitecture. This study adopted BV/TV, TbTh, TbSp, and TbN because they are, according to Bouxsein et al. the four representative indicators of trabecular bone microarchitecture [41]. Similarly, the four parameters of cortical bone were adopted because they have been suggested by previous studies to be representative. Specifically, CtTh and CSA are morphological parameters of the femoral neck; vBMD is a density parameter of the cortical bone of the femoral neck; and BSI reflects the state of health jointly indicated by the morphological parameters and density parameters. Regarding the measurements of bone mineral density, no significant correlation between the aBMD and vBMD was found. The possible reasons leading to this diversity might be the inconsistent measured dimensions, 2D in aBMD or 3D in vBMD, and varied ROI. The grabbed ROI of aBMD from DXA was the proximal femur, including femoral the head and neck, whereas the ROI of vBMD focused on the cortical bone at the femoral neck as in the area shown in Fig. 1.

This study’s results agree with those of studies on the trabecular bone microarchitecture of the femur in ovariectomized rats and normal rats [30, 31]. In the present study, the trabecular bone microarchitecture in the OVX group was more cancellous than that in the control group. However, this study differs from the literature with respect to findings on the parameters of trabecular bone microarchitecture in the femur for the OVX and control groups. This difference was probably due to the differences in the breed and the age of rats as well as in the length of the post-ovariectomy period. More importantly, micro-CT measures different bone regions. Studies have mostly examined the trabecular bone microarchitecture in the distal femoral metaphysis [30, 31], whereas this study examined the cancellous bone structure of the femoral neck. Despite the aforementioned differences, however, this study’s observation that the bone structure of the OVX group was significantly more cancellous than that of the control group are consistent with those of previous studies.

Clinical and animal experiments have suggested that intermittent PTH can (1) treat ovariectomy-induced osteoporosis, (2) improve the bone mineral density of the lumbar vertebra and the femur, (3) improve the microarchitecture of the trabecular bone and cortical bone, and (4) decrease the risk of fractures [42–44]. Using micro-CT, Washimi et al. [31] observed changes in the trabecular bone microarchitecture of the distal femoral metaphysis in ovariectomized rats treated with intermittent PTH; specifically, in their OVX group, intermittent PTH increased BV/TV (by 48.7%), TbN (by 20.5%), and TbTh (by 22.7%) and decreased TbSp (by 24.3%). Such experimental results are similar to those in the present study on the OVX group trabecular bone parameters of the femoral neck. However, in the OVX  +  PTH group of the present study, BV/TV and TbTh were significantly higher (by 24.3% and 21.1%, respectively) compared with the values for the OVX group; TbSp and TbN did not significantly differ. This was probably due to the diverse trabecular bone distribution ratios of different body parts [45] or to the different reactions of various body parts to intermittent PTH treatment; these explanations require verification in future studies. Nonetheless, due to estrogen deficiency, intermittent PTH treatment can improve the trabecular bone loss due to estrogen deficiency of the femoral neck (as indicated by the results for BV/TV and TbTh).

In addition, intermittent PTH treatment can accelerate cortical bone formation in the tibial midshaft as well as increase the area and strength of the cortical bone [46]. A human cadaveric study by Werner et al. suggested that the bone mineral content in the femoral neck only accounted for 23.5% [47] and that the contribution of the cancellous bone to the mechanical strength of the femoral neck was marginal; these findings are attributable to most of the load being borne by the cortical bone [48, 49]. Furthermore, in a previous study, when the cancellous bone in the femoral neck was hollowed, the load to fracture of the femoral neck only decreased by 7% [50]. These studies have revealed that changes in the cortical bone in the femoral neck play an important role in mechanical strength. However, other studies have furnished opposing findings that the cancellous bone is related to femoral neck strength [51, 52]. Evidence on the correlation between cortical bone parameters and femoral neck strength remain somewhat conflicting, and how medical treatment changes the cortical bone and mechanical strength of the femoral neck remain to be clarified. Moreover, due to the particular shape of the femoral neck, its strength is affected not only by bone mineral density, but also by geometric shape and cortical bone microarchitecture, including parameters such as bone surface CSA, cross-sectional moment of inertia, and cortical thickness [53]. The results of this study indicated that although aBMD and the parameters of trabecular bone are not significantly correlated with mechanical strength, the mechanical strength is highly correlated with the CSA and BSI of the cortical bone. Consistent with those of previous studies, this study’s findings indicate the importance of the cortical bone to the femoral neck with respect to mechanical strength. From the statistical analysis, the BSI, which is multiplication of vBMD and CSA, was highly correlated with maximum fracture force of the femoral neck. This positive correlation between BSI and mechanical strength of the femoral neck might be caused by the direct relation of maximum fracture load to CSA, but not vBMD. Furthermore, we also found that the enhanced mechanical strength in the femoral necks of the rats of the OVX  +  PTH group was probably due to the changes produced through intermittent PTH treatment in the cortical bone structure of the femoral neck. Several studies have demonstrated the curative effect of intermittent PTH treatment on osteoporosis of the femoral neck. However, few studies have examined the correlations of intermittent PTH with microstructural changes in the femoral neck and with mechanical strength, and further research on this topic is required.

This study has the following limitations. First, this animal study did not involve human participants. Second, the sample size for each group was five, slightly lower than that in previous studies, in which six bone specimens were included in each group [54–56]. Third, this study did not examine other bone biochemical indices through a blood test; it only used micro-CT scans to estimate bone structural parameters. Fourth, this study did not measure the parameters of other bones with frequent osteoporosis-induced fractures, such as the vertebra body or radius; it only measured the bone structural parameters of the femoral neck.

## Conclusions

Intermittent PTH helped treat ovariectomy-induced osteoporosis of the cancellous bone and cortical bone in the femoral necks of rats. The ability of the femoral neck to resist fracture was highly correlated with two parameters, namely the CSA and BSI of the cortical bone in the femoral neck, and it was less correlated with aBMD or other bone structural parameters.

## Materials and methods

### Animal preparation and experimental design

For this study’s experiment, animal research ethics approval was obtained from the Research Ethics Committee of the Taichung Veterans General Hospital (Permit Number: La1031191). All experiments were conducted in accordance with relevant guidelines and regulations. This study adopted fifteen 8-week-old female Wistar rats, which were divided into three groups of five rats each. These groups were the (1) control group, where the rats were not ovariectomized and were raised to the age of 20 weeks; (2) ovariectomized group (OVX group), where the rats were bilaterally ovariectomized at the age of 8 weeks and raised for the following 12 weeks to simulate postmenopausal osteoporosis in women; and (3) treated group (OVX  +  PTH group), where the rats were bilaterally ovariectomized at the age of 8 weeks, followed by an intermittent treatment with Forteo^®^, a recombinant human PTH with the 34 amino-terminal amino acids, for the following 12 weeks (injection of 50 μg/kg twice weekly). Intermittent PTH injections were performed subcutaneously in those OVX animals of treated group commencing the day after surgery. After the period of 3-month intervention, all the rats were killed at the age of 20 weeks, and their right femur was extracted for further testing. All the removed bones were covered with gauze soaked in 0.9% saline before immediately being preserved in a refrigerator at − 20 °C. We devoted to avoid the bones’ dehydration during the whole experiment. All specimens were kept well after thawing and each of them was separately covered in saline-soaked gauze as well as packed well in zipper storage bags to prevent the dehydration during the whole experiment. These bone specimens were only defrosted to room temperature before the examinations were performed. The extracted bones were subjected to the DXA and micro-CT test first in 1 week and then they were set up to complete biomechanical test in another week. All of the tests were completed in 2 weeks. The collected data were further analyzed.

### Measurement of mineral density of areal bone

Frozen samples were defrosted to room temperature prior to DXA measurement. A single qualified DXA technician conducted all examinations for all samples. All extracted femurs were measured using a fan beam densitometer designed for clinical use (Lunar Prodigy Advanced System, General Electric, Madison, WI, USA), which was calibrated daily according to the manufacturer’s instructions. A DXA machine has two X-ray beams with different energy levels, which are aimed at the target bones. The bone mineral density (BMD) can be calculated based on the absorption of each beam by the bone, while soft tissue absorption is subtracted out. Because the whole femurs of experimental rats were extracted without the enveloping soft tissue, a PMMA plate had to be placed underneath the femoral bone to simulate the density of surrounding soft tissue in a DXA scan, according to the manufacturer’s instructions. All specimens were manually placed on an 8-mm-thick polymethylmethacrylate (PMMA) plate to simulate surrounding soft tissue before being scanned individually per the manufacturer’s recommendation. The scan was set to be in the small animal mode, and a laser beam helped mark the start of each scan. The whole femur was scanned using Prodigy DXA. The aBMD of the region of interest (ROI) of the proximal femur, including the femoral head and the neck proximal to the lowest margin of the lesser trochanter, was analyzed using enCORE software (2007, version 11.20.068).

### Microcomputed tomography scanning and measurements of bone parameters

**Fig. 2:**
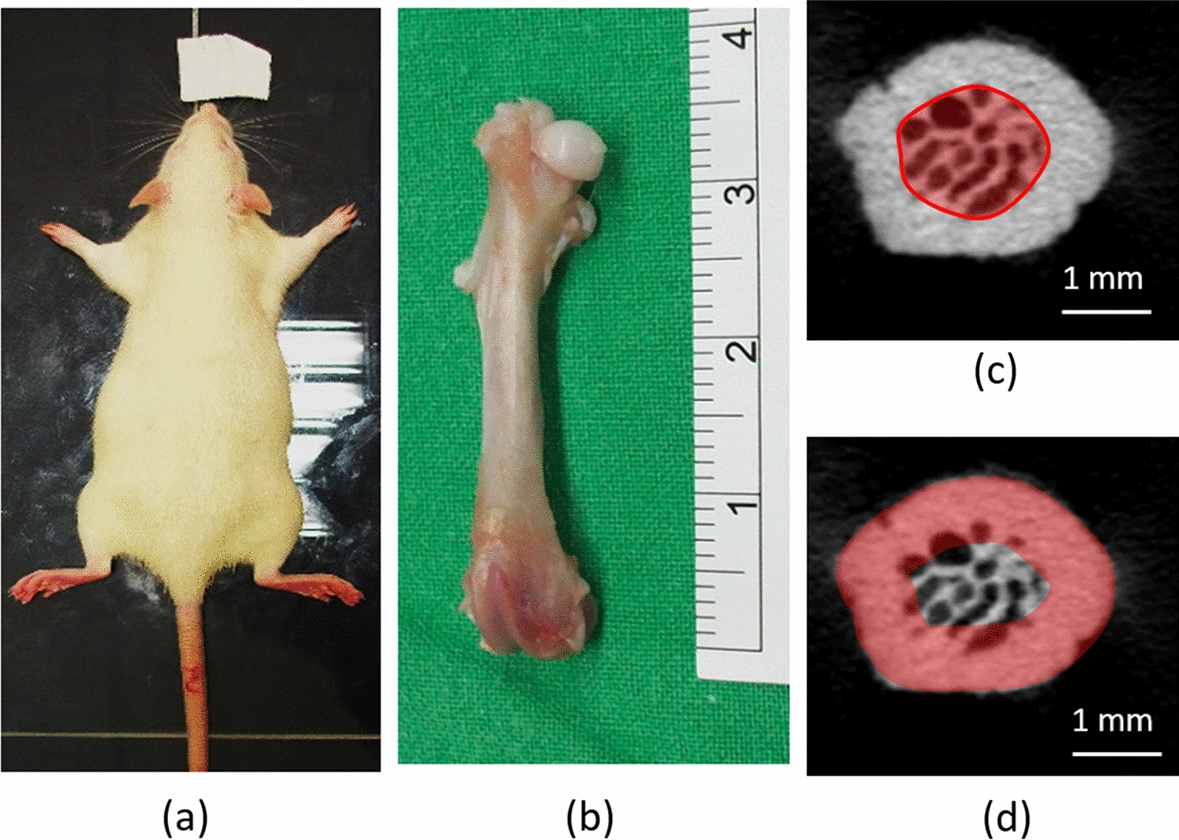
**a** Image of the Wistar female rat used in this study; **b** image of the right femur; **c** ROI of the cancellous bone in the femoral neck from a micro-CT cross section image; **d** ROI of the cortical bone in the femoral neck from a micro-CT cross section image

Frozen samples were defrosted to room temperature prior to the micro-computed tomography (micro-CT) scan. The micro-CT images of each femur were obtained using a Skyscan 1076 micro-CT device (Aartselaar, Belgium; Fig. 2). The scanning parameters were set as follows: tube voltage, 49 kV; current, 200 μA; exposure time, 500 ms; and voxel resolution, 18.27 μm. The micro-CT scanning images were saved in the TIF file format. After scanning was conducted, the images were imported into NRecon (SkyScan, Aartselaar, Belgium) for image reconstruction. The micro-CT images were imported into CTAn software (Skyscan) to measure the cortical bone and trabecular bone parameters of the femoral neck. The global threshold value for obtaining binary images of the mineralized trabecular bone and the ROIs of the cortical bone and cancellous bone were segmented manually. The following four parameters of trabecular bone microstructure in the femoral neck were measured: bone volume fraction (bone volume/total volume, BV/TV, unit: %), trabecular bone thickness (TbTh, unit: mm), trabecular bone separation (TbSp, unit: mm), and trabecular bone number (TbN, unit: mm^−1^) [41, 57]. In addition, the four parameters of the cortical bone in the femoral neck were also measured: cortical bone thickness (CtTh), volumetric bone mineral density (vBMD), cross-sectional area (CSA), and bone strength index (BSI, where BSI  =  vBMD × CSA) [41].

### Femoral neck fracture test

**Fig. 3:**
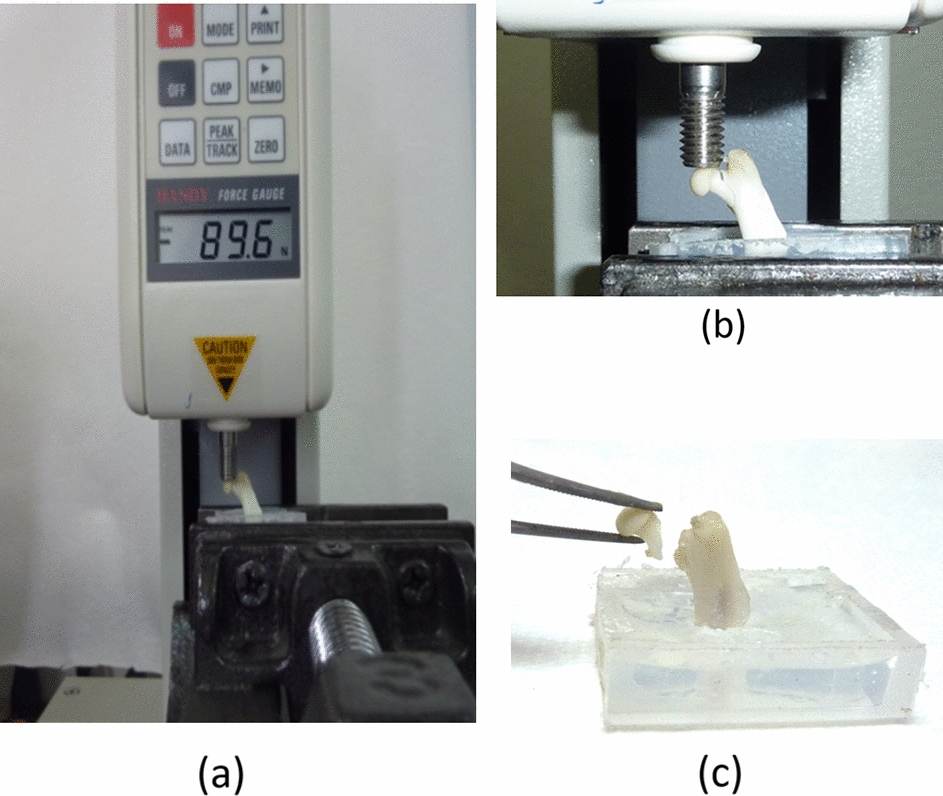
**a** Mechanical strength of the femoral neck measured using a material testing system. **b** Mechanical load was applied downwardly on the femoral head. **c** Femoral neck fracture until maximum fracture force was applied

Pliers were used to cut off the middle section of the femur to obtain the proximal femur. Generally, the proximal femur was retained about 2 cm in length and temporarily fixed vertically in the container. Then the epoxy resin was used to embed and fix approximately 1 cm away from the distal end of the proximal femur [58–60], which was then placed in a material testing system (JSV-H1000, Japan Instrumentation System, Nara, Japan; Fig. 3a). The displacement control mode was used to impose a downward force at a speed of 5 mm/min on the femoral head until the femoral neck fractured (Fig. 3b). The fracture pattern on the femoral neck of all specimens was similar, which was oblique transcervical femoral neck fracture (Fig. 3c). During the experiment, the maximum fracture force was recorded.

### Statistical analysis

The data for aBMD, maximum fracture force of the femoral neck, and cancellous and cortical bone parameters of the femoral neck in the three groups were summarized in terms of the median and interquartile range (IQR). A Kruskal–Wallis test was used to analyze differences in CtTh, BV/TV, TbTh, TbSp, and TbN in the femoral neck between the three groups; Mann–Whitney exact tests were used for post hoc pairwise comparisons; and Spearmen’s correlation coefficients (*r* values) of maximum fracture force with the parameters for the femoral neck and bone structure (aBMD, CtTh, vBMD, CSA, BSA, BV/TV, TbTh, TbSp, and TbN) were calculated. All statistical analyses were performed using SPSS Version 19 (IBM Corporation, Armonk, NY, USA). The level of the statistical significance was set at *p*  < 0.05.

## Data Availability

All data generated or analyzed during this study are included in this published article.

## Abbreviations

pths: Parathyroid hormones
OVX: Ovariectomized
DXA: Dual-energy X-ray absorptiometry
abmd: Areal bone mineral density
ctth: Cortical bone thickness
vbmd: Volumetric bone mineral density
CSA: Cross-sectional area
BSI: Bone strength index
BV/TV: Bone volume fraction
tbth: Trabecular thickness
tbsp: Trabecular separation
tbn: Trabecular number
PMMA: Polymethylmethacrylate
micro-CT: Micro-computed tomography
